# Enhanced Photocatalytic Activity of Anatase/Rutile Heterojunctions by Lanthanum and Tin Co-Doping

**DOI:** 10.3390/ijms231911339

**Published:** 2022-09-26

**Authors:** Xiaodong Zhu, Fengqiu Qin, Lili He, Yu Jiao, Wei Feng

**Affiliations:** 1 School of Mechanical Engineering, Chengdu University, Chengdu 610106, China; 2 School of Science, Xichang University, Xichang 615013, China

**Keywords:** anatase/rutile heterojunctions, sol–gel method, La/Sn co-doping, photocatalytic activity

## Abstract

Anatase/rutile heterojunctions were prepared using the sol–gel method and modified by La/Sn single doping and co-doping. Sn doping promoted the transformation from anatase to rutile, while La doping inhibited the phase transformation. La and Sn co-doping showed an inhibitory effect. The co-doping of La and Sn did not increase visible-light absorption, but exhibited a synergistic effect on inhibiting the recombination of photogenerated electrons and holes, which improved the photocatalytic activity on the basis of single-element modification. The first-order reaction rate constant of La/Sn co-doped sample was 0.027 min^−1^, which is 1.8 times higher than that of pure TiO_2_ (0.015 min^−1^). Meanwhile, the mechanism of photodegradation of methylene blue (MB) by La/Sn co-doped anatase/rutile heterojunctions was discussed through electrochemical measurements and free-radical trapping experiments.

## 1. Introduction

As a photocatalytic material, TiO_2_ is favored in the field of photocatalytic degradation of pollutants due to its advantages of stable chemical properties, nontoxicity, low cost, availability, and reusability [1,2,3]. The photogenerated electrons and holes of pure TiO_2_ are easily recombined, which limits its practical application. Modifying TiO_2_ by ion doping can form lattice defects, change the energy band structure, inhibit the recombination of photogenerated electrons and holes, and improve the photocatalytic efficiency [4,5,6,7]. Due to the synergistic effect of different elements, the co-doping modification is known to be more efficient than single doping in enhancing the photocatalytic performances of functional materials [8,9,10,11,12,13,14]. Chen et al. [15] loaded Ce/N co-doped TiO_2_ on diatomite, and the results showed that Ce/N co-doped sample had the smallest bandgap width because N doping made the valence band move up, and Ce doping formed an impurity level in the forbidden band, which were conducive to narrowing the bandgap width. Furthermore, the Ce/N co-doped sample showed the smallest grain size and the largest specific surface area. The activity of the co-doped sample was higher than that of the single-doped samples.

It has been reported that the photocatalytic activity of TiO_2_ is improved obviously after La doping [16,17,18,19]. Nesic et al. [16] and Peng et al. [17] found that the specific surface area of TiO_2_ increased after La doping, which was conducive to the photocatalytic activity. The study of Xin et al. [18] showed that La doping contributed to the increase in anatase content, the formation of the Ti–O–La bond was conducive to the improvement of adsorption performance, and the introduced O vacancies effectively inhibited the recombination of carriers, thus showing higher photocatalytic activity than pure TiO_2_. On the other hand, Sn-doped TiO_2_ exhibited better photocatalytic performance than pure TiO_2_ [20,21,22,23]. Mohamed et al. [20] found that, after Sn doping, the specific surface area increased, the visible-light absorption significantly improved, and the recombination of photogenerated electrons and holes was inhibited, which were advantageous to the photocatalytic activity. Therefore, it is reasonable to speculate that the La/Sn co-doping modification of TiO_2_ can give play to the advantages of each element, form a synergistic effect, and achieve a better modification effect.

In this work, pure TiO_2_ with anatase and rutile mixed crystal was prepared using the sol–gel method and then modified by single doping and co-doping of La and Sn elements, respectively. Pure TiO_2_, Sn-doped TiO_2_, La-doped TiO_2_, and La/Sn co-doped TiO_2_ were labeled as PT, ST, LT, and LST, respectively. The crystal structure, surface morphology, elemental composition, valence state, and optical properties of samples were analyzed, and the mechanism of improving the photocatalytic performance of co-doped TiO_2_ was systematically discussed.

## 2. Results and Discussion

### 2.1. Photocatalyst Characterization

**Figure 1:**
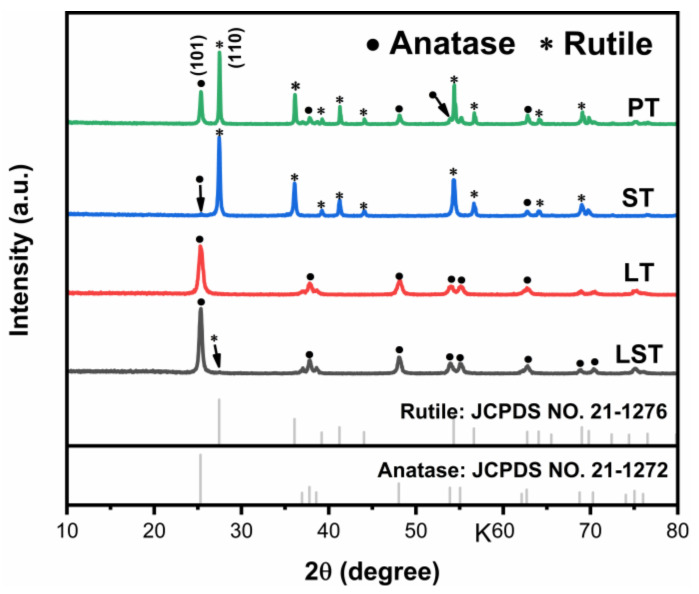
XRD patterns of samples.

Figure 1 shows the XRD patterns of samples. The diffraction peaks of PT pattern appeared near 27.3°, 41.2°, and 44.3°, corresponding to the crystal planes of rutile (110), (111), and (210), respectively (JCPDS NO. 21-1276). The diffraction peaks of anatase (101), (004), and (200) crystal planes appeared at 25.3°, 37.8°, and 48.1° (JCPDS NO. 21-1272). The results show that PT had an anatase/rutile mixed crystalline structure. Most of the diffraction peaks corresponded to rutile phase in the ST pattern, indicating that Sn doping promoted the transformation from anatase to rutile. Correspondingly, anatase peaks could only be found in the pattern of LT, suggesting that the phase transformation was inhibited by La doping. Due to their similar ionic radius, Sn^4+^ ions (0.069 nm) could enter the lattice to replace Ti^4+^ ions (0.0605 nm), causing lattice defects, weakening the binding force between atoms, and making the Ti–O bonds easier to crack, which was conducive to phase transition [5]. Furthermore, since SnO_2_ and rutile have the same crystal structure, SnO_2_ would become the heterogeneous nucleation center of rutile, promoting the transformation [22]. The radius of La^3+^ ions (0.103 nm) is much larger than that of Ti^4+^ ions, hindering their entry into the TiO_2_ lattice. La elements should be dispersed on the surface of TiO_2_ particles in the form of oxide La_2_O_3_ [6]. The transformation requires the rearrangement of atoms and the breaking of bonds. La_2_O_3_ dispersed on TiO_2_ surface will hinder the migration of Ti and O atoms, and delay the nucleation and growth of rutile, inhibiting the phase transition [6,19]. It can be seen that the peaks in LST were indexed to anatase, and only a weak peak at 27.3° ascribed to rutile was detected, implying that LST was composed of mostly anatase and a small amount of rutile, revealing that the inhibition of phase transformation by La doping was stronger than the promotion of Sn doping.

**Figure 2:**
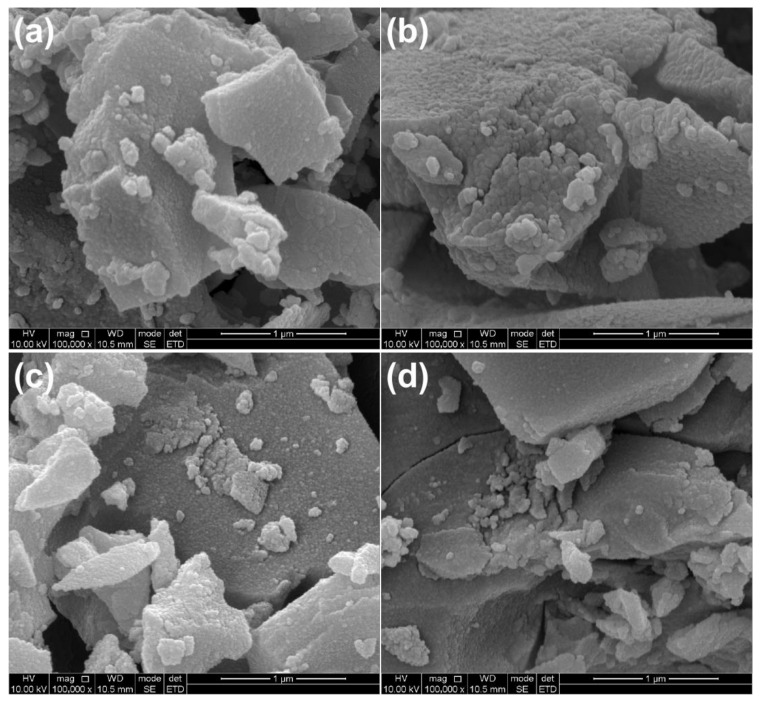
SEM images of PT (**a**), ST (**b**), LT (**c**), and LST (**d**).

Figure 2 presents the SEM images of samples. It can be observed that PT was composed of agglomerates with different shapes, and the size distribution of agglomerates ranged from tens to hundreds of nanometers. ST, LT, and LST samples were also composed of agglomerates of different shapes. No obvious change in particle morphology can be found in SEM images before and after doping.

**Figure 3:**
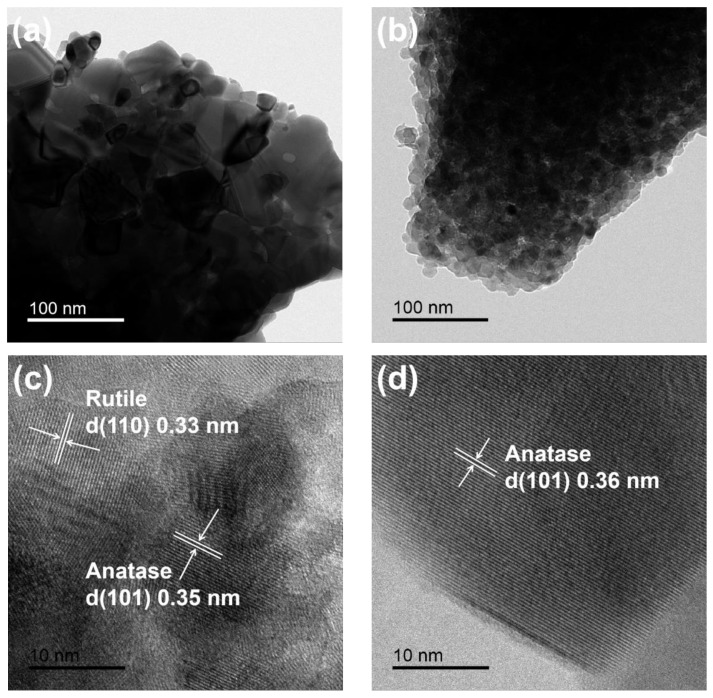

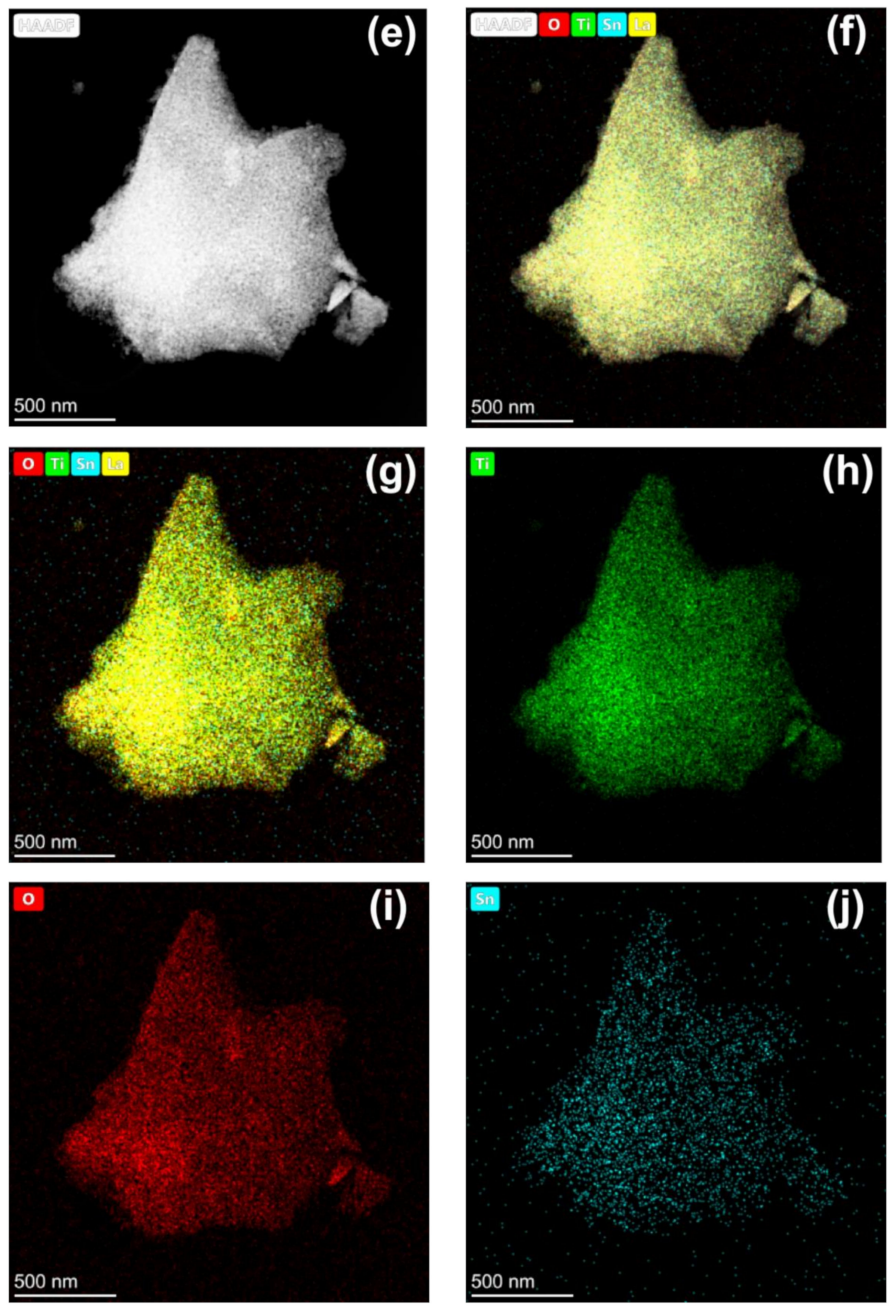

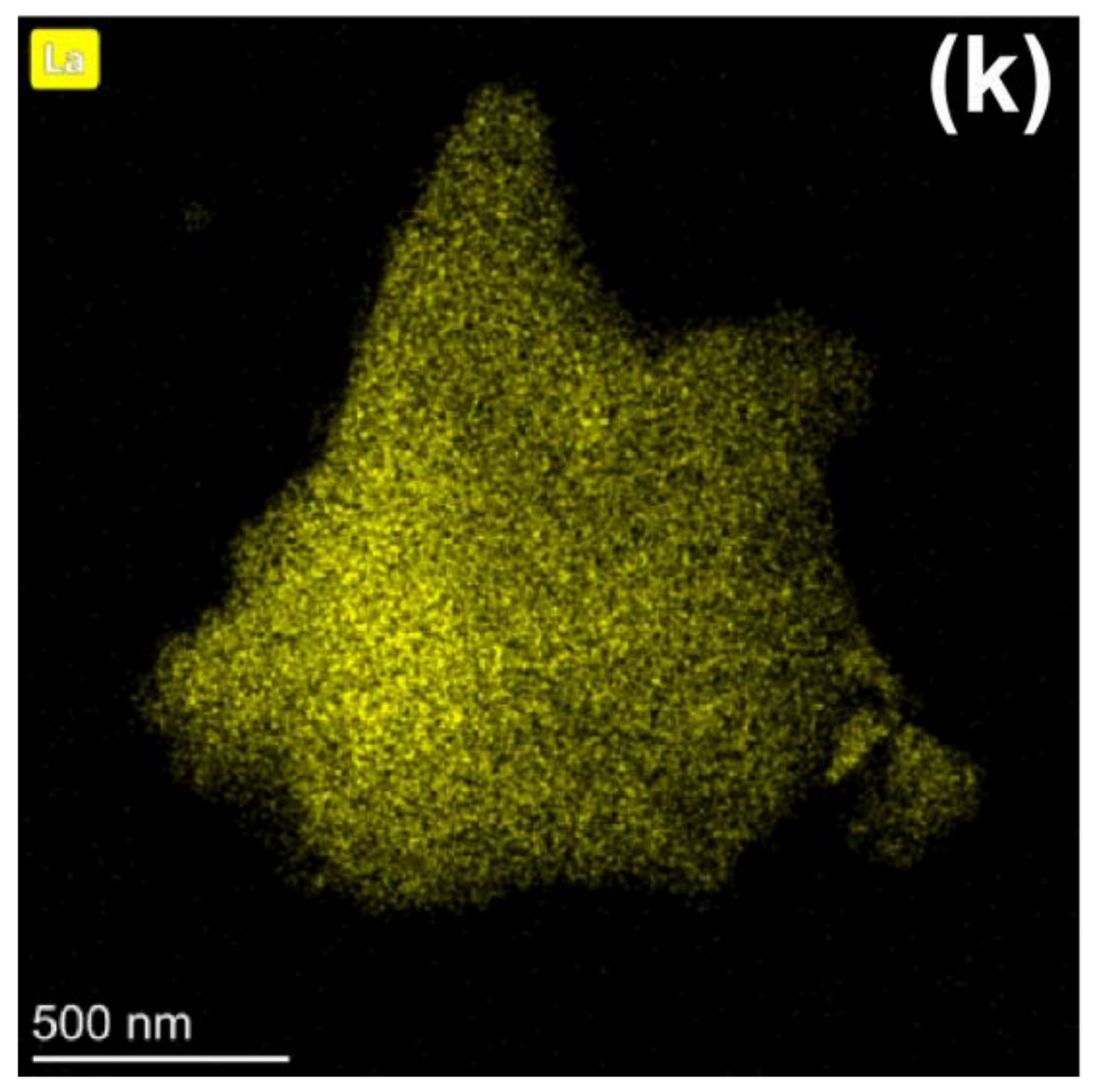
TEM and HRTEM images of PT (**a**,**c**) and LST (**b**,**d**), and the STEM mappings of LST (**e**–**k**).

Figure 3 shows the TEM images of PT (a) and LST (b). It can be observed that PT particles were seriously agglomerated, and it was difficult to distinguish the size of single particles. LST particles were also agglomerated, possibly due to the high temperature of 600 °C, and the particles grew larger during the process of heating. The HRTEM images of PT (c) and LST (d) are shown in Figure 3c,d. The crystal lattice stripes were clear, indicating that the samples had good crystallinity. The spacings of crystal planes marked in Figure 3c were 0.35 nm and 0.33 nm, corresponding to the anatase (101) crystal plane and the rutile (110) crystal plane, respectively [24,25,26,27], indicating that PT was a mixed crystal composed of anatase and rutile, consistent with the XRD results. The crystal plane spacing 0.36 nm marked in Figure 3d was indexed to the anatase (101) crystal plane of LST. The signals of Ti, O, La, and Sn elements could be found in the STEM mappings of LST (Figure 3e–k), indicating that La and Sn elements already existed in TiO_2_ due to doping, and the four elements were substantially uniformly distributed in the LST sample.

**Figure 4:**
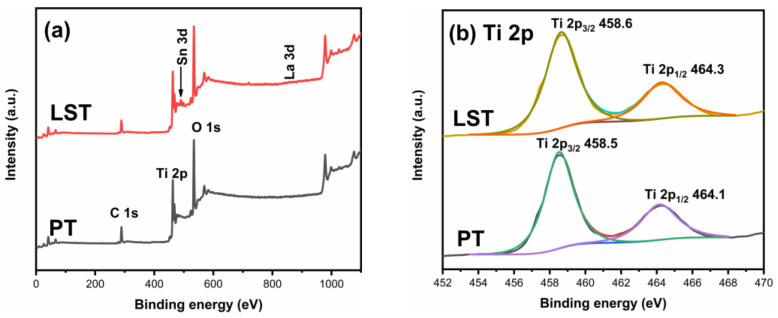

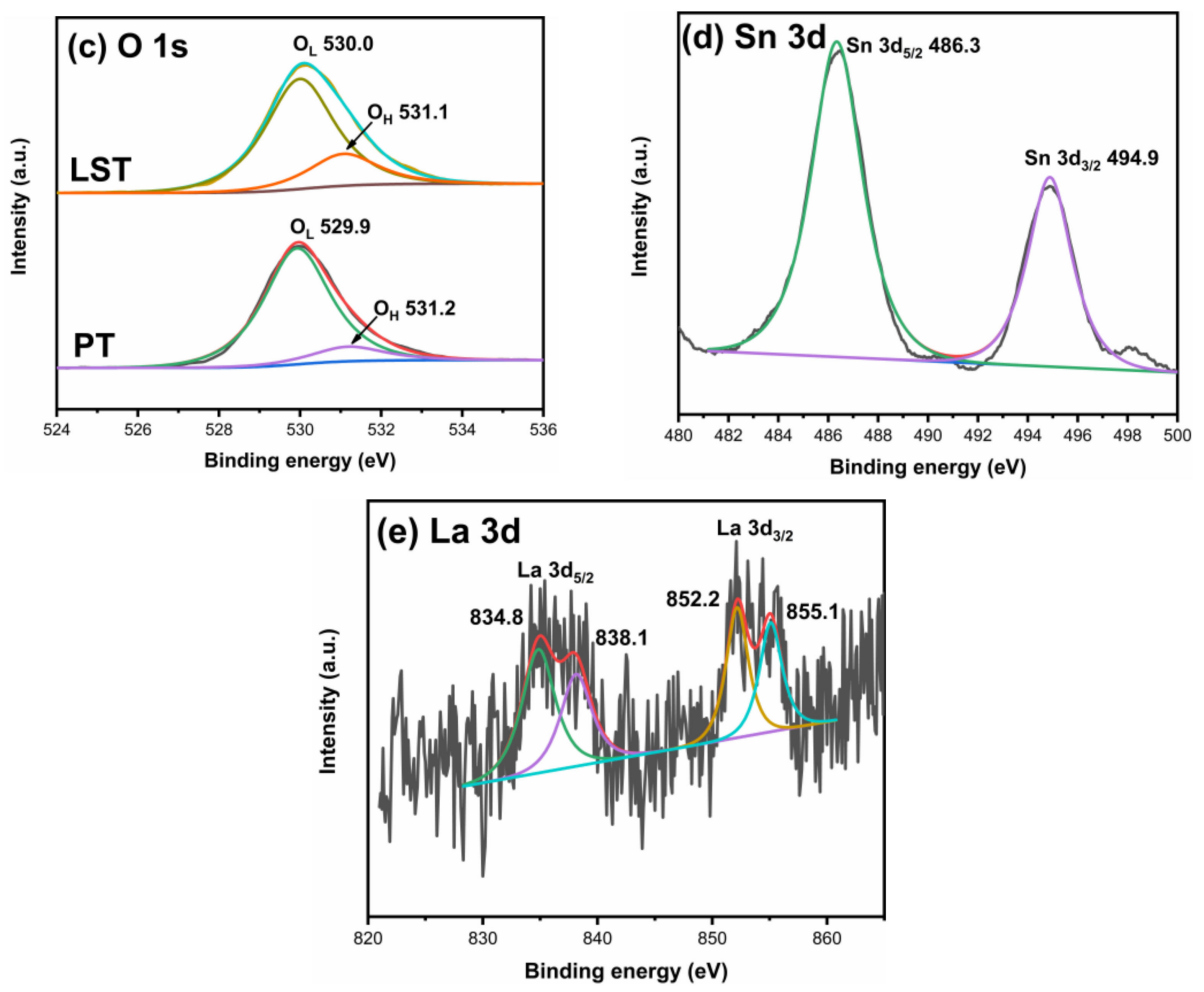
XPS survey of PT and LST (**a**); high resolution spectra of Ti 2p (**b**), O 1s (**c**), Sn 3d (**d**), and La 3d (**e**).

Figure 4 presents the XPS spectra of PT and LST. The appearance of the La 3d peak and Sn 3d peak in the LST spectra confirms the existence of La and Sn elements in the co-doped sample. Figure 4b shows the high-resolution spectra of Ti 2p. The Ti 2p of PT was decomposed into two peaks, located at 458.5 eV and 464.1 eV, corresponding to Ti 2p_3/2_ and Ti 2p_1/2_, indicating that the valence state of Ti was +4. The two peaks corresponding to Ti 2p_3/2_ and Ti 2p_1/2_ were located at 458.6 eV and 464.3 eV in LST, indicating that Ti in the co-doped sample also had a valence of +4 [15,28,29]. The high-resolution spectra of O 1s are shown in Figure 4c. The O 1s of PT was decomposed into two peaks corresponding to the lattice oxygen (O_L_) and surface hydroxyl (O_H_), located at 529.9 eV and 531.2 eV, respectively. The peaks of the lattice oxygen and surface hydroxyl of LST sample were at 530.0 eV and 531.1 eV respectively [30]. It can be observed that the surface hydroxyl peak area of the LST sample was significantly larger than that of PT, indicating that the co-doping advanced the surface adsorption performance and introduced more OH^−^ groups on the particle surface. The percentages of oxygen in the surface hydroxyl group of PT and LST were 12.1% and 22.6%, respectively, indicating that co-doping could enhance the surface hydroxyl content. This is beneficial to generate more hydroxyl radical and improve photocatalytic performance during photodegradation [31]. The Sn 3d high-resolution spectrum of LST is shown in Figure 4d. The two peaks at 486.3 eV and 494.9 eV corresponded to Sn 3d_5/2_ and Sn 3d_3/2_, respectively. The location of these two peaks indicates that the Sn element had a +4 valence [21,23,32]. The high-resolution spectrum of La 3d is shown in Figure 4e. The peaks at 834.8 eV and 838.1 eV corresponded to La 3d_5/2_, and the peaks at 852.2 eV and 855.1 eV corresponded to La 3d_3/2_, indicating that the La element existed in the form of La^3+^ [31,33].

**Figure 5:**
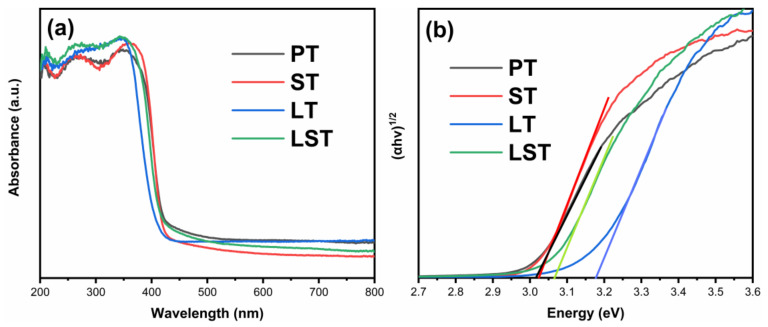
UV/Vis absorption spectra (**a**) and bandgap energy (**b**) of samples.

Figure 5 shows the UV/Vis absorption spectra (a) and the bandgap (b) of the samples. In the ultraviolet region, all samples had high absorption, whereas, in the visible region, absorption decreased rapidly. The absorption edge of ST was almost the same as that of PT, and the absorption edges of LT and LST showed a weak blue shift. The bandgaps of PT, ST, LT, and LST were 3.02, 3.02, 3.17, and 3.06 eV, respectively. It has been documented that the absorption edge of TiO_2_ red-shifts and the bandgap decreases after Sn or La doping [31,34,35]. However, in this work, the bandgaps of LT and LST increased slightly. Combined with XRD, it can be seen that the transformation from anatase to rutile was inhibited by La doping. Due to the bandgap of anatase (3.2 eV) being larger than that of rutile (3.0 eV), the bandgaps of LT and LST samples increased compared to PT.

**Figure 6:**
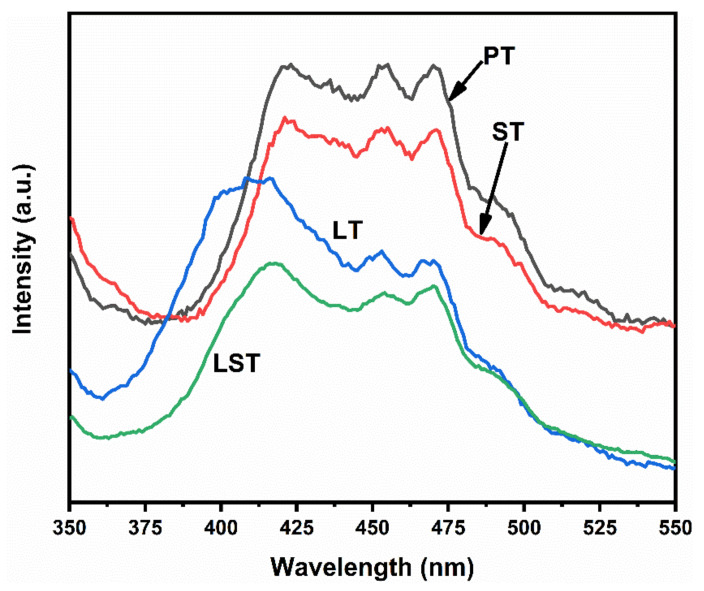
PL spectra of samples.

The PL spectra of samples are shown in Figure 6. PT and LST showed the same spectral shape but different intensity. The PL peak intensity decreased after Sn doping, indicating that the recombination of photogenerated electrons and holes was inhibited. The main PL peaks of PT and ST appeared at approximately 422 nm, due to the photons released when the photogenerated electrons returned directly from the conduction band to the valence band [32]. DRS results show that the bandgap of PT and ST was 3.02 eV, and the corresponding photon wavelength was 410 nm, which was about 12 nm less than the corresponding wavelength of 422 nm in the main peak of PL. This deviation could be attributed to the Stokes shift [36,37]. Compared with PT, the PL main peaks of LT and LST shifted to a lower wavelength, consistent with the bandgaps of LT and LST being larger than that of PT. The PL peak intensity of LT was lower than that of PT, indicating that La doping could also inhibit the recombination of photogenerated carriers. In particular, the PL peak intensity of LST was the lowest, implying that La/Sn co-doping had a synergistic effect on retarding the recombination of photogenerated charges, which was beneficial to the photocatalytic performance.

### 2.2. Photocatalytic Performance

**Figure 7:**
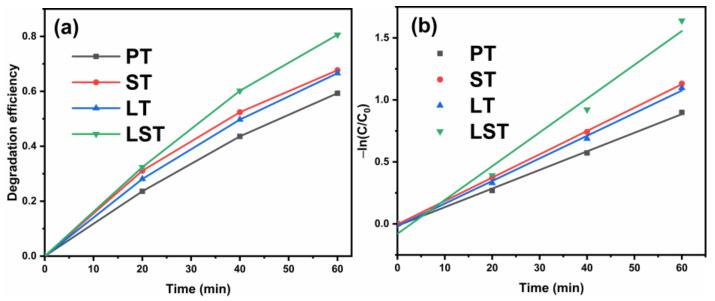
Degradation curves (100 mL of MB (10 mg/L) degraded by 0.05 g of sample at pH = 7 and room temperature of 25 °C) (**a**) and kinetics curves (**b**) of samples.

Figure 7 shows the MB degradation degree curves (a) and kinetics curves (b) of samples. After 60 min, the degradation degree of PT was 59.3%, and the degradation degrees of ST, LT, and LST were 67.7%, 66.6%, and 80.6%, respectively. The degradation of MB on the surface of TiO_2_ photocatalyst conformed to a first-order reaction, and the reaction rate constant k was calculated using the formula kt = −ln(C/C_0_), where C_0_ and C are the initial concentration of MB solution and the concentration at time t, respectively. A larger k value denotes a faster reaction rate and a better photocatalytic activity. The first-order reaction rate constants of PT, ST, LT, and LST were 0.015, 0.019, 0.018, and 0.027 min^−1^, respectively. The first-order reaction rate constants of ST, LT, and LST are 1.3, 1.2, and 1.8 times higher than that of PT, respectively, indicating that the reaction rate was accelerated after doping. Table 1 summarizes the degradation degrees of reported photocatalysts.

### 2.3. Photocatalytic Degradation Mechanism

**Figure 8:**
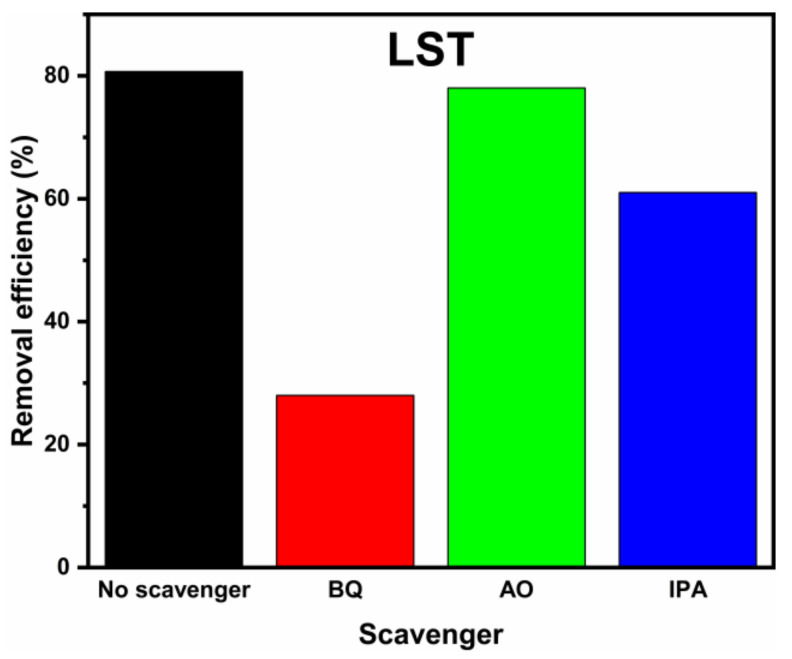
Degradation degrees of LST in the presence of different scavengers.

Figure 8 shows the results active species experiments of LST. When benzoquinone (BQ), isopropanol (IPA), and ammonium oxalate (AO) were added as the radical scavengers, the degradation degrees of LST decreased from 80.6% to 28.1%, 61.5%, and 78.0%, respectively. Since BQ, IPA, and AO are capture agents of •O_2_^−^, •OH, and photogenerated holes (h^+^) [44,45], the results show that the •O_2_^−^ radical was the main active group, whereas h^+^ and •OH played a subsidiary role in the degradation process toward MB.

**Figure 9:**
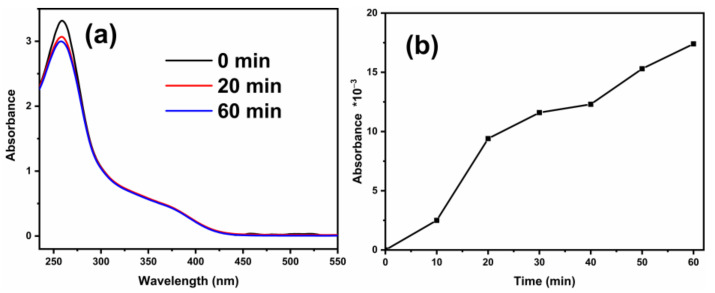
Absorbance curves of NBT (**a**) and 2,3-HBA (**b**) of LST.

Nitro-blue tetrazolium (NBT) and 2,3-dihydroxybenzoic acid (2,3-HBA) experiments were launched to further verify that the •O_2_^−^ and •OH radicals were yielded in the degradation process, and the results are shown in Figure 9. The presence of •O_2_^−^ radicals can be testified by the reaction of NBT with •O_2_^−^ to form a purple precipitate. With the extension of illumination time, the •O_2_^−^ radicals react with NBT to generate more and more purple precipitates, thus consuming NBT and gradually reducing its absorbance. On the other hand, salicylic acid (SA) reacts with •OH radicals to form 2,3-HBA which has a special absorption at 510 nm [44,45]. With the increase in time, the absorbance of NBT decreased and the absorbance of 2,3-HBA increased, indicating that •O_2_^−^ radicals and •OH radicals were generated in the system.

**Figure 10:**
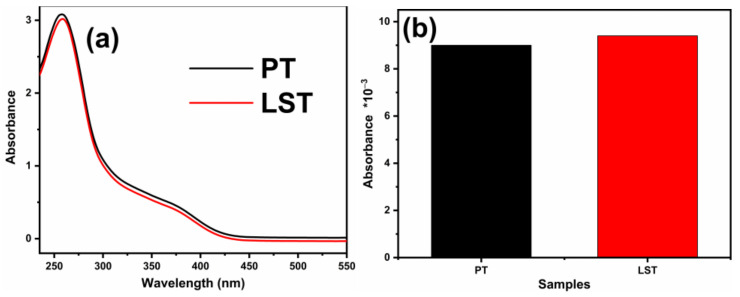
NBT absorbance (**a**) and 2,3-HBA (**b**) absorbance of LST and PT after 20 min.

Figure 10 shows the NBT and 2,3-HBA absorbance of LST and PT after illumination for 20 min. The NBT absorbance of PT was higher than that of LST, indicating that LST generated more •O_2_^−^ radicals. The 2,3-HBA absorbance of PT was lower than that of LST, proving that LST produced more •OH radicals. During the photodegradation process, LST generated more free radicals, implying that it had a higher photogenerated charge separation rate compared to PT, consistent with the results of the PL spectra and photocatalytic activity experiments.

**Figure 11:**
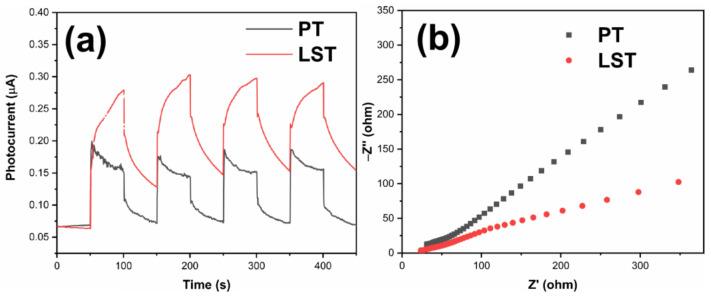
Photocurrent responses curves (**a**) and electrochemical impedance spectroscopy (**b**) of PT and LST.

The separation and transfer of photogenerated charges of PT and LST were further studied through photoelectrochemical measurements. Figure 11a shows the photocurrent responses curves (PC) of PT and LST. Generally, a higher photocurrent density denotes a higher separation efficiency of photogenerated charges [46,47]. Both PT and LST generated photocurrent under light, and the photocurrent density of LST was higher than that of PT, indicating that Sn and La co-doping was beneficial to improve the separation efficiency of carriers. Figure 11b shows the electrochemical impedance spectra (EIS) of PT and LST. According to Nyquist’s theorem [46,48,49], the arc radius of LST was smaller than that of PT, indicating that LST had a lower charge motion resistance and a better electron mobility.

**Figure 12:**
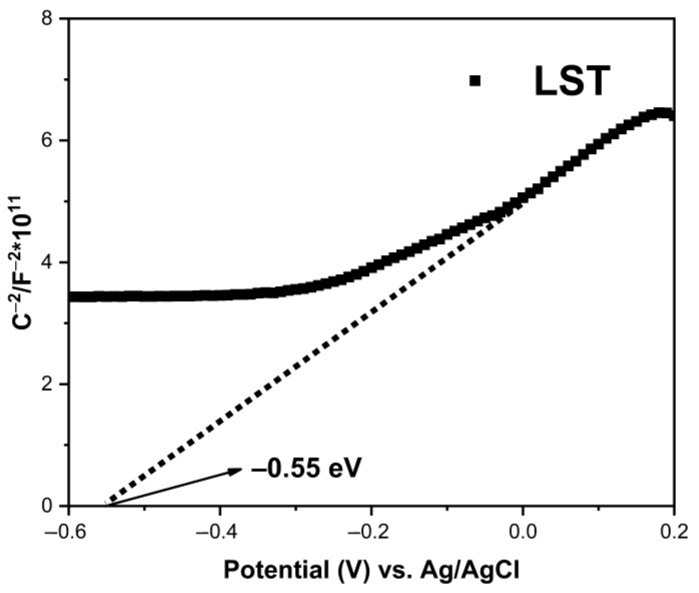
Mott–Schottky plot of LST.

Figure 12 shows the Schottky curves of PT and LST. The electrode/electrolyte was is measured according to the Mott–Schottky equation (1) [48,50,51].

(1) $$\frac{1}{C^{2}}=\frac{2}{N_{D}e\varepsilon_{0}\varepsilon }\left(E-E_{FB}-\frac{kT}{e}\right),$$

where *C* is the space charge capacitance in the semiconductor, *N_D_* is the electron carrier density, *e* is the elemental charge, *ε_0_* is the permittivity of a vacuum, *ε* is the relative permittivity of the semiconductor, *E* is the applied potential, *E_FB_* is the flat band potential, *T* is the temperature, and *k* is the Boltzmann constant. The slope of the linear part of the curve was positive, indicating that LST is an n-type semiconductor [48,49,51]. In Equation (1), if 1/C^2^ = 0, the *E_FB_* value of the LST flat band potential could be estimated to be −0.55 V vs. Ag/AgCl. According to the formula E_NHE_ = E_Ag/AgCl_ + 0.197 [52,53,54], the E_FB_ of LST could be calculated to be about −0.35 V vs. NHE. It is generally believed that the CB bottom (E_CB_) is about 0.1 V more negative than the potential of E_FB_ for n-type semiconductors [55,56]. Therefore, the E_CB_ of LST could be determined to be −0.45 V vs. NHE. According to the formula E_VB_ = E_g_ + E_CB_ [49,51], the potential of VB (E_VB_) of LST could be calculated to be +2.61 V vs. NHE.

**Figure 13:**
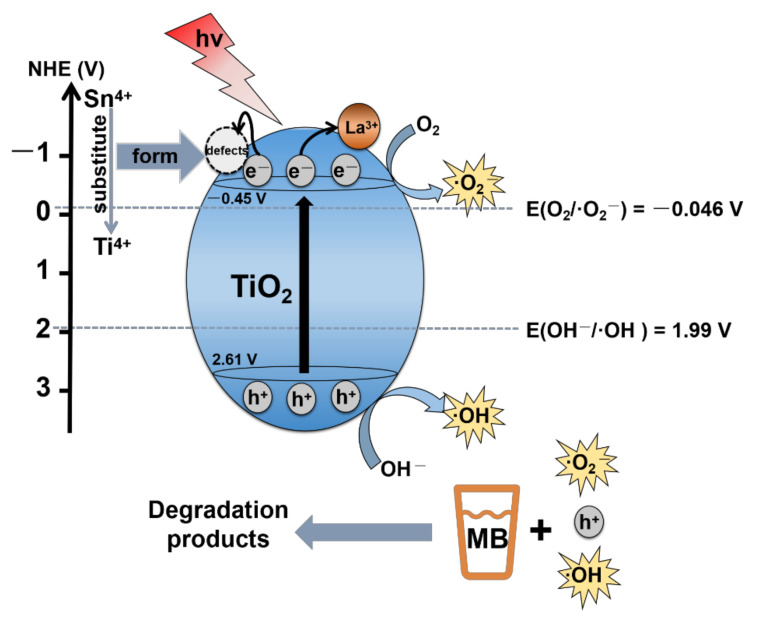
Schematic diagram of MB photodegradation by LST.

Some studies reported that ion doping brings impurity energy levels below the conduction band of TiO_2_, reduces the bandgap width, and increases the absorption of light [20,23]. Conversely, several studies also showed that there is a blue shift after doping [57,58]. In this work, DRS results show that the bandgap width of PT was 3.02 eV and that of LST was 3.06 eV, without a red shift, which could be ascribed to the fact that co-doping of Sn and La inhibited the transformation from anatase to rutile, and the forbidden band width of rutile was lower than that of anatase. Consequently, a weak blue shift occurred after La/Sn co-doping, implying that the utilization of visible light did not improve via co-doping. It can be seen from PL spectra that single doping of Sn and La or co-doping could inhibit the recombination of photogenerated electrons and holes, and LST exhibited the best inhibition ability. According to the DRS and Mott–Schottky results, the E_CB_ of LST was −0.45 V, which is higher than E_0_(O_2_/•O_2_^−^) (−0.046 V vs. NHE) [59,60]. The potential position of CB ensured the generation of •O_2_^−^ radicals, as also confirmed by NBT experiments. On the basis of the above results, the mechanism diagram of photodegradation of MB by LST is shown in Figure 13.

On one hand, as the radius of Sn^4+^ is close to that of Ti^4+^, the replacement of Ti^4+^ by Sn^4+^ ions would form crystal defects, capture photogenerated charges, and improve quantum efficiency [61,62,63]. On the other hand, no peak related to La element was detected in the XRD pattern. The radius of La^3+^ ions is much larger than that of Ti^4+^ ions; thus, the possibility that La^3+^ ions entered the TiO_2_ lattice can be excluded. Instead they were dispersed on the surface of TiO_2_ particles in the form of oxide La_2_O_3_, which captured photogenerated charges, improving the separation of carriers [16,33,64]. Therefore, the co-doping of Sn and La produced a synergistic effect on improving the carrier separation; as a result, its photocatalytic activity was higher than that of single-element doping in PT.

## 3. Materials and Methods

Butyl titanate (Analytical Reagent, AR, ≥98.0%), anhydrous ethanol (AR, ≥99.7%), glacial acetic acid (AR, ≥99.5%), tin tetrachloride pentahydrate (AR, ≥99.0%), lanthanum nitrate hexahydrate (AR, ≥99.0%), benzoquinone (AR, ≥98.5%), ammonium oxalate (AR, ≥99.5%), and isopropanol (AR, ≥99.7%) were purchased from Chengdu Chron Chemicals Co., Ltd., (Chengdu, China).

### 3.1. Sample Preparation

Butyl titanate and anhydrous ethanol were added into the beaker at a volume ratio of 1:2 to form solution A. Deionized water, glacial acetic acid and anhydrous ethanol were mixed in a volume ratio of 2:3:7.5 to form solution B. After being evenly stirred, solution B was dropped into solution A, which was continuously stirred to form sol and then aged. After the aging, the gel was dried in the oven, and finally heat-treated at 600 °C. After grinding, pure TiO_2_ powder was obtained, labeled as PT. Certain amounts of SnCl_4_·5H_2_O and La(NO_3_)_3_·6H_2_O were added to solution B, and the other steps were the same to prepare the doped sample with a Sn/Ti molar ratio of 3% and a La/Ti molar ratio of 0.5%. Sn/La co-doped TiO_2_ could be prepared by adding SnCl_4_·5H_2_O and La(NO_3_)_3_·6H_2_O into solution B, where the molar ratio of Sn/Ti was 3% and the molar ratio of La/Ti was 0.5%. Sn-doped TiO_2_, La-doped TiO_2_, and La/Sn co-doped TiO_2_ were labeled as ST, LT, and LST, respectively.

### 3.2. Sample Characterization

The crystal structure of the samples was analyzed using a DX-2700 X-ray diffractometer (Dandong Haoyuan Instrument Co. Ltd., Dandong, China, XRD). An FEI-Inspect F50 scanning electron microscope (FEI Company, Hillsboro, OR, USA, SEM) and an FEI-Tecnai G2 F20 transmission electron microscope (FEI Company, Hillsboro, OR, USA, TEM and HRTEM) were used to observe the morphology. An XSAM800 multifunctional surface analysis system was used to analyze the element composition and valence state of samples (Kratos Ltd., Manchester, UK, XPS). A UV-3600 ultraviolet/visible-light spectrophotometer was used to study the optical absorption performance (Shimadzu Group Company, Kyoto, Japan, DRS). Detection of the recombination of photogenerated electrons and holes was investigated using an F-4600 fluorescence spectrometer (Shimadzu Group Company, Kyoto, Japan, PL). The photocurrent response curves, electrochemical impedance spectroscopy, and Mott–Schottky plots were measured using a DH-7000 electrochemical workstation (Jiangsu Donghua Analytical Instrument Co., Ltd., China, PC, EIS and MS).

### 3.3. Photocatalysis Experiment

The photocatalytic activity of the sample was evaluated with MB solution as the target pollutant. Briefly, 100 mL of MB (10 mg/L) solution and 0.05 g of sample powder were mixed at room temperature (25 °C) and kept neutral. After stirring for 30 min in the dark, the mixture was irradiated using a 250 W xenon lamp (300–800 nm). Samples were taken every 20 min. The absorbance of the supernatant was tested at 664 nm. The MB degradation degree was calculated using the formula (A_0_ − A_t_)/A_0_ × 100%, where A_0_ and A_t_ are the initial and time t absorbances of MB.

On the basis of the MB degradation system, 2 mL (0.1 mol/L) of benzoquinone (BQ, •O_2_^−^ trapping agent), isopropanol (IPA, •OH trapping agent), and ammonium oxalate (AO, h^+^ trapping agent) were added to investigate the active species.

## 4. Conclusions

Pure TiO_2_ was prepared using the sol–gel method and modified by La/Sn single doping and co-doping. PT had an anatase/rutile mixed crystalline structure, Sn promoted the transformation from anatase to rutile, and La inhibited the transformation. The inhibition of La was stronger than that of Sn, and the phase transformation from anatase to rutile was inhibited by co-doping. La/Sn co-doping did not produce an obvious red shift; however, the content of hydroxyl on the surface of TiO_2_ particles increased, and a synergistic effect was produced on inhibiting the recombination of photogenerated electrons and holes. The results of electrochemical experiments also showed that the separation and transfer of photogenerated charges were faster after La/Sn co-doping, and the quantum efficiency was improved. Therefore, the photocatalytic activity of LST was superior to that of ST, LT, and PT. The first-order reaction rate constant of PT was 0.015 min^−^^1^, and the first-order reaction rate constants of ST, LT, and LST were 1.3, 1.2, and 1.8 times higher than that of PT, respectively. The active species experiments of LST showed that •O_2_^−^ radicals were the main active groups during the photodegradation process.

## Figures and Tables

**Table 1:** The summarization of degradation degrees by various photocatalysts.

Ref.	Method	Photocatalyst	Light Source	Target Pollutant	Decolorization Degree
[29]	Sol–gel method	Fe–TiO_2_	UV light (15 W)	RB 69 (100 mg/L)	98.0% in 120 min
[31]	Sol–gel method	La–N–TiO_2_/diatomite	Xenon lamp (150 W)	RhB (10 mg/L)	93.0% in 240 min
[38]	Combustion synthesis methodology	Mg_2_Si(Si)/MgO	LED lamp (100 W)	MB (50 mg/L)	90.0% in 120 min
[39]	Ball-milling/molten salt processing approach	Co_3_Fe_7_/CoFe_2_O_4_@ carbon	LED lamp (100 W)	MB (100 mg/L)	100% in 12 min
[40]	Acid-treatment method	Mg_2_Si	LED lamp (100 W)	MO (50 ppm)	100% in 30 min
[41]	Hydrothermal method	SDBS–TiO_2_	Xenon lamp (500 W)	RhB (10 mg/L)	90.0% in 120 min
[42]	Hydrothermal method	Ag–TiO_2_	Xenon lamp (500 W)	RhB (20 mg/L)	80.0% in 240 min
[43]	Electrospinning method	Fe–ZnO	Mercury lamp	MB (10 mg/L)	88.0% in 360 min
[44]	Solvothermal method	BiOI/Bi_2_SiO_5_	Xenon lamp (500 W)	MO (10 ppm)	70.0% in 360 min
This work	Sol–gel method	La–Sn–TiO_2_	Xenon lamp (250 W)	MB (10 mg/L)	80.6% in 60 min

## Data Availability

Not applicable.
